# Special Issue “Role of Redox Homeostasis and Oxidative Stress in Human Health”

**DOI:** 10.3390/ijms24098352

**Published:** 2023-05-06

**Authors:** Hung-Yao Ho

**Affiliations:** 1 Metabolomics Core Laboratory, Healthy Aging Research Center, Chang Gung University, Taoyuan City 33302, Taiwan; hoh01@mail.cgu.edu.tw; Tel.: +886-3-2118800 (ext. 3318); 2 Clinical Metabolomics Core Laboratory, Chang Gung Memorial Hospital, Taoyuan City 33302, Taiwan; 3 Department of Medical Biotechnology and Laboratory Science, College of Medicine, Chang Gung University, Taoyuan City 33302, Taiwan; 4 Research Center for Emerging Viral Infections, Chang Gung University, Taoyuan City 33302, Taiwan

Redox homeostasis plays essential roles in the regulation of the physiological process. When disrupted, it is implicated in the pathogenesis of degenerative diseases, such as diabetes mellitus, Alzheimer’s disease, atherosclerosis and cardiovascular diseases, and even infectious diseases. At the molecular level, reactive species, for instance, nitric oxide, superoxide, hydrogen sulfide, and carbon monoxide, can act as regulatory and signaling molecules themselves. Some of these reactive species oxidize certain cysteine and methionine residues of target proteins to alter their structures and functions. Some of these proteins are enzymes whose activity changes usher in the reprogramming of metabolism. Other proteins serve as signaling molecules, for example, kinases, which transduce signal upon oxidation. Conversely, some transducers, once activated, generate reactive oxygen and nitrogen species. The molecular aspects of the involvement of reactive species in health and disease pathogenesis and the underlying mechanism are largely unexplored. Clinically, the biomolecules that undergo oxidation serve as biomarkers for diseases. On the other hand, antioxidants that diminish the reactive-oxygen- and nitrogen-species-induced damages exert biological effects. This Special Issue consists of four reviews and four original articles that contribute significantly to our knowledge of redox biology and medicine. 

Čižmárová et al. described the general aspects of redox homeostasis in the body and in a special body fluid–saliva [1]. Redox homeostasis and the salivary antioxidant system are discussed in terms of health and disease conditions, such as dental caries, periodontal diseases, oral cancers, cardiovascular diseases, pancreatitis, diabetes mellitus, and systemic sclerosis. Further work in this research field may yield diagnostic or prognostic biomarkers for these diseases. The inter-relationships between oxidative stress, signal transduction, and cellular responses are depicted in reviews and original articles. Santibáñez-Andrade et al. discussed how particulate matter (PM) elicits oxidative stress and molecular signaling events, such as the oxidation of proteins, DNA, and lipids, leading to multiple carcinogenesis-related processes [2]. The latter cellular processes include proliferation, evasion of tumor-suppression mechanisms, anti-apoptosis mechanism, angiogenesis induction, and metastasis. The genotoxicity and tumorigenicity of PM in air pollutants and the involvement of reactie oxygen and nitrogen species in their toxic effects are discussed in this review. Additionally, Pecchillo Cimmino et al. detailed the effect of NADPH oxidase (NOX)-generated superoxide anion on various metabolic pathways, with particular reference to the NOX1-, NOX2-, and NOX4-induced reprogramming of the cancer cell metabolism [3]. Ortiz-García reported that NOX2 and NOX4 interact and form aggregates in the acrosome of mammalian spermatozoa [4]. The interaction is disrupted in the capacitated spermatozoa in a calpain-dependent manner. NOX2 and NOX4 initiate the generation of reactive oxygen species, which has a functional correlation in spermatozoan physiology. Saeed et al. also reported that mitogen-activated protein kinase 8 interacting protein-1 (MAPK8IP1) is involved in reactive oxygen species generation, inflammasome activation, and apoptosis initiation in palmitate-treated INS-1 cells [5]. These findings imply that MAPK8IP1 acts upstream of the reactive-oxygen-species-mediated signaling pathway. 

Impaired redox homeostasis and increased oxidative stress are related to the pathogenesis of human diseases. Greeck et al. gave a detailed account of the metabolic and mitochondrial dysfunction in the immune cells of multiple sclerosis (MS) patients and lymphocytes of the MS model mice [6]. Lymphocytic oxidative stress is intimately associated with metabolic switching and the alteration of functions in immune cells. Oxidative damage markers can be biomarkers for human diseases. Serum levels of carbonyl groups were elevated in acute ischemic stroke (AIS) patients, while the urinary levels of melatonin metabolite were lowered in them [7]. These parameters are suggestive of increased oxidative stress and a reduced antioxidant defense in these patients. The pharmacological activities of pharmaceuticals can be related to their redox activities. Koszelewski et al. reported the synthesis of *p*-quinols with antimicrobial activities against pathogenic *E. coli* strains [8]. Interestingly, such antimicrobial activities correlated with their DNA oxidation activities. 

These studies contribute to our knowledge of redox homeostasis and its applied aspects in human health and disease. 
